# Impact of Insecticide-Treated Net Ownership on All-Cause Child Mortality in Malawi, 2006–2010

**DOI:** 10.4269/ajtmh.15-0929

**Published:** 2017-09-27

**Authors:** Lia S. Florey, Adam Bennett, Christine L. Hershey, Achuyt Bhattarai, Carrie F. Nielsen, Doreen Ali, Misheck Luhanga, Cameron Taylor, Thomas P. Eisele, Yazoume Yé

**Affiliations:** 1 ICF, The DHS Program, Rockville, Maryland;; 2 Malaria Elimination Initiative, University of California, San Francisco, California;; 3 President’s Malaria Initiative, United States Agency for International Development, Arlington, Virginia;; 4 President’s Malaria Initiative, Malaria Branch, Centers for Disease Control and Prevention, Atlanta, Georgia;; 5 National Malaria Control Programme, Ministry of Health, Lilongwe, Malawi;; 6 Center for Applied Malaria Research and Evaluation, School of Public Health and Tropical Medicine, Tulane University, New Orleans, Louisiana;; 7 ICF, MEASURE Evaluation, Rockville, Maryland

## Abstract

Insecticide-treated nets (ITNs) have been shown to be highly effective at reducing malaria morbidity and mortality in children. However, there are limited studies that assess the association between increasing ITN coverage and child mortality over time, at the national level, and under programmatic conditions. Two analytic approaches were used to examine this association: a retrospective cohort analysis of individual children and a district-level ecologic analysis. To evaluate the association between household ITN ownership and all-cause child mortality (ACCM) at the individual level, data from the 2010 Demographic and Health Survey (DHS) were modeled in a Cox proportional hazards framework while controlling for numerous environmental, household, and individual confounders through the use of exact matching. To evaluate population-level association between ITN ownership and ACCM between 2006 and 2010, program ITN distribution data and mortality data from the 2006 Multiple Indicator Cluster Survey and the 2010 DHS were aggregated at the district level and modeled using negative binomial regression. In the Cox model controlling for household, child and maternal health factors, children between 1 and 59 months in households owning an ITN had significantly lower mortality compared with those without an ITN (hazard ratio = 0.75, 95% confidence interval [CI] = 0.62–90). In the district-level model, higher ITN ownership was significantly associated with lower ACCM (incidence rate ratio = 0.77; 95% CI = 0.60–0.98). These findings suggest that increasing ITN ownership may have contributed to the decline in ACCM during 2006–2010 in Malawi and represent a novel use of district-level data from nationally representative surveys.

## INTRODUCTION

The entire population of Malawi, 16.4 million, is at risk of *Plasmodium falciparum* malaria infection, which remains a significant threat to health and child survival. According to best available estimates from the National Statistics Office of Malawi, malaria is responsible for a substantial proportion of health-care consumption, accounting for 29% of all outpatient visits and approximately 40% of hospital deaths and 30% of deaths in children less than 5 years of age.^1,2^ To address this high burden of disease, global partners have contributed substantial funds to malaria control efforts, including provision of commodities such as insecticide-treated nets (ITNs) and antimalarial drugs. Indoor residual spraying (IRS) of insecticides is another effective intervention funded by international donors and governments. Due to its cost, IRS is currently in limited use. Distribution of ITNs for household use is the major malaria vector control intervention in Malawi. Between 2004 and 2010, two-thirds of the funding for malaria commodities contributed by the Global Fund to Fight AIDS, Tuberculosis, and Malaria (Global Fund) and the President’s Malaria Initiative (PMI) procured ITNs.^3^

Randomized controlled trials (RCTs) have demonstrated an 18% protective efficacy of ITNs in reducing child mortality.^4^ Despite the proven protective efficacy under controlled conditions, ITN benefits under routine conditions may be mitigated by poor quality of nets due to wear or decreasing insecticidal properties, or due to nonuse or improper use. Investigating the protective effects of ITNs in real-world settings is methodologically challenging due to limitations of the available data. Many malaria-endemic countries do not have sufficiently robust vital registration or routine health information systems to produce reliable malaria-specific mortality estimates. Protective effects of ITNs on child mortality estimated from studies using observational data have ranged from 44% in a longitudinal cohort study in Kenya,^5^ to 27% in a case control study from a demographic surveillance system in Tanzania (both among children 1–59 months),^6^ and 25–38% in a longitudinal analysis of surveillance data in The Gambia (among children 1–9 years).^7^ Lim and others used nationally representative household survey data in a multicountry analysis and found a 23% reduction in mortality among children 1–59 months of age.^8^

National-level evaluations of ITN impact using cross-sectional survey data are not well suited to assessing causal associations and are subject to confounding. In cross-sectional survey data reverse associations, in which areas of high malaria transmission (and high malaria mortality) are targeted for ITN distribution, may confound analysis of impact. In addition, although single surveys may provide valid estimates of child mortality, they may not be powered to show changes in mortality due to interventions. However, in the absence of reliable data on cause-specific mortality from vital registration systems, survey data are valuable resources for national-level evaluations, despite their limitations.

One possible methodological approach to overcome the constraints of survey data is to pool data across surveys to maximize power and apply matching techniques to minimize bias from unmeasured confounding factors.^8^ Other methodological approaches that have been used in impact evaluations include before and after assessments (also known as adequacy designs), comparison of program and nonprogram areas, regression discontinuity, decomposition analysis, and evaluation platform designs. Victora and Bryce suggest the possibility of using a district platform where adequate subnational data are available.^9^ Nationally representative household surveys in Malawi provide a unique opportunity to examine the impact ITN ownership has had on child health, as multiple surveys were powered at the district level. Graves and others used a similar approach to investigate the relationship between malaria control interventions, climate, and incident malaria cases in *subzoba* (districts) in Eritrea.^10^ Similarly, Bennett and others conducted a dose–response ecological analysis with district-months as the unit of analysis to evaluate the association between ITN program intensity and outpatient malaria case incidence using routine program data from Zambia.^11^ Finally, a recently published case study of under-five mortality in Malawi estimated that ITNs were responsible for 20% of the observed reduction in under-five mortality between 2000 and 2013 using a district-level decomposition analysis (the Lives Saved Tool).^12^

This study used two distinct analytic approaches to evaluate the impact of increasing ITN ownership on all-cause under-five mortality in Malawi between 2006 and 2010. The first approach used cross-sectional survey data to construct a retrospective cohort and applied an individual-level Cox proportional hazards model to examine the relationship between ITN ownership and deaths in children under five. A second, ecological, analysis was carried out in which assignment of risk factors (ITN coverage) and outcomes (under-five deaths) was at the district level. The ecological analysis made use of the very large sample size of the 2010 Demographic and Health Surveys (DHS) in Malawi, with 24,825 households included, allowing data on ITN coverage and of other covariates to be aggregated and powered for results at the district level.

## METHODS

### Data sources.

Household survey data from DHS Program were primarily used for this study. Demographic and Health Surveys were conducted in Malawi in 2000, 2004, and 2010.^13–15^ In addition, a Multiple Indicator Cluster Survey (MICS) was conducted in 2006 under the direction of United Nations Children’s Fund.^16^ Each of these surveys used a two-stage stratified sample to collect nationally representative data on sociodemographic and health indicators, including child mortality, child and maternal health interventions, and individual and household characteristics. The sampling frame for the surveys was determined based on enumeration areas from national censuses. Survey primary sampling units (PSUs/clusters) were selected with a probability proportional to population size within domains and households were selected with equal probability systematic sampling within clusters. Within a selected household all women between the ages of 15 and 49 were asked to complete an interview in which they were asked about their health and the health of their children born in the past 5 years. In addition, a full birth history was completed in which interviewed women were asked to provide date of birth and age at death for all children ever born. Finally, these surveys collected information on malaria-specific indicators including ownership and use of ITNs.

In addition to national survey data, this study also used data on *P. falciparum* prevalence estimates among children 2 to 10 years of age (*Pf*PR_2–10_) for 2010 from the Malaria Atlas Project.^17^ Rainfall and temperature data from the Famine Early Warning System^18^ and Moderate Resolution Imaging Spectroradiometer,^19^ respectively, were also incorporated at the cluster level.

### Statistical analyses.

#### Individual-level model.

An individual-level model was developed to look at the association between ITN ownership and mortality in children 1–59 months of age using data from the 2010 DHS. Although it would be preferable to use data on ITN use by individual children as the exposure variable, the available survey data does not contain information on retrospective ITN use. However, the relationship between ITN use and ITN ownership is fairly linear; nonuse of ITNs is largely determined by lack of access.^20,21^

##### Dataset assembly.

This analysis used birth histories of women interviewed for the 2010 Malawi DHS to construct a retrospective cohort of children for a 3-year period before the survey. The primary outcome measure was deaths in children less than 5 years of age during this period. The primary exposure of interest was ITN ownership which was derived from questions in the household questionnaire on bednet ownership, type of net, insecticide treatment of net, and duration of ownership. To identify an individual child’s exposure to an ITN, data on the duration of ownership of ITNs and the time of retreatment of nets (if any) was used to construct a time-varying indicator of ITN ownership for 3 years before the survey. Other time-varying covariates such as high malaria transmission season (December–May), child’s age, mother’s age, *P. falciparum* prevalence estimates among children 2–10 years of age (*Pf*PR_2–10_), rainfall and minimum temperature were constructed. The age variables were constructed retrospectively using the child’s and mother’s reported ages from the time of the interview. *Pf*PR_2–10,_ rainfall and temperature lagged by 2 months were linked to DHS cluster locations using global positioning system (GPS) coordinates at cluster centroids. Other covariates of interest were included as static estimates from the time of interview. These include residence (urban or rural), household wealth quintile (based on a principal component analysis [PCA] of household assets), parity, mother’s education, and household water source. Cluster-level DPT3 immunization coverage (the percent of children 12–23 months of age with at least three doses of DPT immunization), cluster-level diarrhea prevalence (percent of children under five with diarrhea in the 2 weeks before the survey), and cluster-level coverage of skilled birth attendance (the percent of births in the past 5 years that were attended by a skilled health professional) were constructed for inclusion in models.

##### Matching.

If successful, matching mitigates confounding within nonexperimental study designs to allow causal inference to be drawn between exposure (ITN ownership) and an expected outcome (child deaths).^22^ Eligible children living in households with ITNs and those without were matched into strata based on key confounding factors. This preprocessing of data was done at the group level, and not by individuals, so no observations were dropped. Exact matching was conducted on collapsed attributes related to the child including household wealth (above/below median PCA score), urban/rural household residence, cluster-level *Pf*PR_2–10_ (≥ 40% versus < 40%), DPT3 coverage at cluster-level (above/below median), and mother’s education (secondary+, primary, none) to reduce confounding, as has been recommended previously^8^ (Table 1). Exact matching was conducted using the *MatchIt* package (version 2.4-21) in the statistical package R.^23^

**Table 1:** Basic information on variables included in the Cox Proportional Hazards model

Variable	Description
Matching variables	
Residence	Residence of the household (urban/rural)
Household wealth	Above or below median principal component score for household assets
Education level	Mother's educational level: secondary+, primary or none
Malaria transmission risk	Malaria transmission risk based on MAP 2010 *Pf*PR_2–10_, categorized as < 40% and 40%+
DPT3 Immunization status	Above or below the median PSU-level coverage of three doses of DPT in children 12–23 months of age.
Other variables controlled for in the model	
Household wealth	Survey-specific quintile of household wealth based on household assets (1–5)
Child age category	In months: 1–5, 6–11, 12–23, 24–35, 36–47, 48–59
Mother’s age category	In years: 15–19, 20–24, 25–29, 30–34, 35–39, 40–44, 45–49
Parity	Number of births (continuous)
DPT3 Immunization coverage	PSU-level coverage of 3 doses of DPT in children 12–23 months of age (continuous)
Diarrhea prevalence	PSU-level diarrhea prevalence in children less than 5 years of age in the 2 weeks before survey (continuous)
Season	High transmission season (December–May) for each month of observation
Malaria transmission risk	Malaria transmission risk based on MAP 2010 *Pf*PR_2–10_, categorized as < 40% and 40%+
Rainfall (lagged 2 months)	PSU-level monthly rainfall from FEWS
Minimum temperature (lagged 2 months)	PSU-level monthly minimum temperature from MODIS
Skilled birth attendance	PSU-level skilled birth attendance rate
Water source	Household water source: improved or nonimproved
Malaria control intervention variable	
Monthly ITN ownership	Household owns at least one ITN by month. Constructed using data on ownership of nets, type of net, treatment of net and duration of ownership (monthly up to 36 months before the survey).

FEWS = Famine Early Warning System; MAP = Malaria Atlas Project; MODIS = Moderate Resolution Imaging Spectroradiometer; *Pf*PR_2–10_ = *Plasmodium falciparum* prevalence rate in children age 2–10 years.

##### Statistical analysis.

The relationship between household ITN ownership and child mortality (deaths of children age 1–59 months) over the 36 months preceding the survey was assessed with Cox proportional hazards models using the matched data. The analysis time was measured in months since birth and matched strata were included as a shared frailty, which is the equivalent of a random effect to account for unobserved heterogeneity. The model was further adjusted for several time-varying covariates: season (high malaria transmission season December–May), child’s age (categorical), mother’s age (categorical), rainfall (lagged 2 months) and minimum temperature (lagged 2 months); and for several other covariates including cluster-level DPT3 immunization coverage (among children 12–23 months), cluster-level diarrhea prevalence (in the 2 weeks before the survey among children less than 5 years of age), household wealth quintile, and parity (Table 1). Rainfall and minimum temperature lags were used due to previously documented lagged relationships between climate variables and clinical incidence.^11,24^ The household water source (improved or not) and the cluster-level coverage of skilled birth attendance were omitted from final models due to nonsignificance. Additionally, separate models were compared for rural and urban areas, respectively, the effect of ITN age was explored through a model with ITN ownership categorized by time since the ITN was obtained (greater than or less than 18 months [1.5 years]), and child age (less than or equal to 3 years of age versus older than 3 years of age) was evaluated as a moderator of the effect of ITN ownership through a model including an interaction term. Finally, the association between the number of ITNs owned and child mortality was assessed in a model restricted to only those households with at least one ITN.

#### District-level model.

The association between ITN ownership and child mortality was also assessed using a district-level model in which data were aggregated by district and by year using data from the DHS and the Malawi 2006 MICS. These two surveys are some of the few nationally representative surveys powered to provide estimates of malaria indicators and other child health indicators at the district-level.

##### Dataset assembly.

The outcome of interest was the number of under-five deaths per district per year which was estimated retrospectively from the birth histories collected in the 2010 DHS (see Supplemental Table 1). Two models were developed with different primary exposure variables measuring ITN coverage. First, district-level average household ownership of ITNs was used as the primary exposure variable. Cross-sectional data from the 2006 MICS and the 2010 DHS were used to construct retrospective, district-specific estimates of ITN ownership for survey years. Weighted averages of district-level means for ITN ownership were constructed for interim years between surveys to produce annual estimates. Overall, ITN ownership data from 27 districts were available from 2006 and 2010. For interpretation purposes, district-year average ITN ownership values were categorized into binary values (high or low) based on whether the district-year values were above or below the grand mean.

The second model used modeled ITN ownership from a combination of survey data and administrative records of numbers of ITNs distributed as the primary exposure variable. Data on annual, district-level ITN distribution were used and a decay factor was applied as per previously published methodology.^25^ This decay factor accounts for some loss of ITNs per year (either due to attrition or physical deterioration) and includes ITNs distributed in previous years to estimate total annual ITN ownership. ITN distribution data were also adjusted for midyear district-level population available through census data. Finally, the values were included with ITN ownership data from the 2010 DHS and the 2006 MICS in Loess regression models to create best-fit district-year estimates of ITN ownership.

In addition, data on other sociodemographic and child and maternal health variables were needed for these models. Table 2 provides a description of the individual- and household-level variables that were aggregated at the district-level for modeling. The choice of variables was directed by literature on child survival and data available. As was done in estimating ITN ownership, cross-sectional data from the 2006 MICS and the 2010 DHS were used to construct retrospective, district-level coverage estimates of variables for survey years. Weighted averages of district-level means for these variables were constructed for interim years between surveys. Overall, data from 27 districts were available from 2006 and 2010. For interpretation purposes, retrospective, district-year average values for each variable were categorized into binary values (high or low) based on whether the values were above or below the grand mean. Cut-off values are presented in Table 2.

**Table 2:** Basic information on variables included in district-level models

Variable	Description
Sociodemographic variables	
Residence	Percent of children in urban households (low/high): cut-off 9.7%
Household wealth	Survey-specific quintile of household wealth based on household assets (1–5)
Education level	Percent of children whose mothers attended primary school or more: (low/high): cut-off 79%
Water source	Percent of children with access to improved water source (low/high): cut-off 77%
Sanitation facilities	Percent of children with access to improved sanitation (low/high): cut-off 1.8%
Malaria transmission risk	District-level malaria transmission risk based on MAP 2010 *Pf*PR_2–10_ (low/high): cut-off 41%
Rainfall anomaly	District-level difference between mean annual rainfall and average nine year rainfall (2002–2010) (low/high): cut-off 12.6
Maternal and child health intervention variables	
Tetanus immunization status	Percent of children whose mothers had at least two tetanus injections during last pregnancy ending in live birth in past two years (low/high): cut-off 66%
Immunization status	Percent of children 6–23 months of age with complete immunization (3 doses polio, three doses DPT, BCG, and measles) (low/high): cut-off 62%
Vitamin A supplementation	Percent of children 6–59 months given vitamin A supplement in the past 6 months (low/high): cut-off 70%
Childhood illness variables	
Diarrhea	Percent of children less than 5 years of age had diarrhea in the 2 weeks before survey (low/high): cut-off 21%
Stunting	Percent of children less than 5 years of age greater than two standard deviations below the mean height for age (low/high): 46%
Malaria control intervention variables	
ITN coverage	Percent of children living in a household covered by an ITN according to modeled estimates combining household ITN ownership and ITN distribution data adjusted for population and a lag factor (low/high): 52.4%
ITN ownership	Percent of children living in a household that owns at least one ITN (low/high): 50%

ITN = insecticide-treated net; MAP = Malaria Atlas Project; *Pf*PR_2–10_ = *Plasmodium falciparum* prevalence rate in children age 2–10 years.

In addition to these individual and household-level variables, district-level estimates of malaria transmission risk (*Pf*PR_2–10_) and annual district-level rainfall anomalies were linked to DHS cluster locations using GPS coordinates of the clusters centroids for inclusion in the dataset. As was done with the other variables, the values were dichotomized into high and low categories based on the average district-level value. Cut-off values are presented in Table 2.

##### Statistical analysis.

Generalized linear models with negative binomial distributions were developed to assess associations between aggregated estimates of covariates at the district-year-level and numbers of deaths in children less than 5 years of age per district per year. These models were adjusted for robust clustering at the district-level to account for temporal correlation of the data. Model fit was assessed using the Bayesian Information Criterion. Poisson models were over-dispersed thus multivariable generalized linear models with negative binomial distributions and a person-month offset were used. Stata 13 (Stata Corporation, College Station, TX) was used for all analyses.

## RESULTS

### Individual-level analyses.

Data from the 2010 DHS were used to create a retrospective cohort which consisted of 29,492 children 1–59 months age who contributed 652,775 child-months of person-time. Among these children there were 821 deaths over the observation period. Child-months of observation time and numbers of deaths in children 1–59 months stratified by household ITN ownership status are summarized in Table 3. The crude mortality rate per 1,000 child-months in children from households owning no ITNs was 1.28 compared with 1.20 in children from households with at least one ITN.

**Table 3:** Descriptive bivariate tabulations of 1–59 month mortality by matching strata and household ITN status

Matching strata	No ITN in household			Household has at least 1 ITN		
	Deaths 1–59 months	Child-month time	Crude mortality rate per 1,000	Deaths 1–59 months	Child-month time	Crude mortality rate per 1,000
Wealth						
High	283	231,218	1.22	100	84,887	1.18
Low	347	262,745	1.32	91	73,925	1.23
Region						
Urban	62	48,067	1.29	10	15,601	0.64
Rural	568	445,896	1.27	181	143,211	1.26
*Pf*PR_2–10_						
< 40%	250	241,139	1.04	87	78,177	1.11
≥ 40%	380	252,824	1.50	104	80,635	1.29
DPT3 coverage						
High	301	246,746	1.22	97	81,377	1.19
Low	329	247,217	1.33	94	77,435	1.21
Mother’s education						
Secondary+	63	59,682	1.06	25	25,700	0.97
Primary	435	339,688	1.28	137	109,691	1.25
None	132	94,593	1.40	29	23,421	1.24
Total	630	493,963	1.28	191	158,812	1.20

ITN = insecticide-treated net; *Pf*PR_2–10_ = *Plasmodium falciparum* prevalence rate in children age 2–10 years.

**Figure 1.**
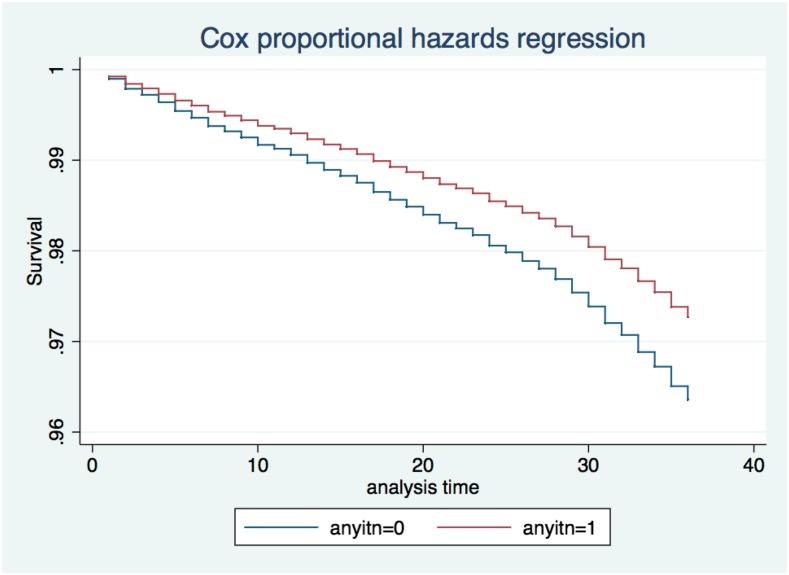
Hazard function by household insecticide-treated net status.

In the matched, multivariable Cox regression models children in households with at least one ITN were significantly less likely to die compared with children from households without ITNs (hazard ratio [HR] = 0.75, 95% confidence interval [CI] = 0.62–0.90; Table 4, Figure 1). Children from the wealthiest households (highest wealth quintile) and children 12 months and older had significantly lower mortality risk than children from the least wealthy households and those 1–5 months of age. Mother’s age, parity, diarrhea prevalence in children, coverage of three DPT immunizations, *Pf*PR_2–10_, season, rainfall, and minimum temperature did not have a significant effect on child survival in adjusted models.

**Table 4:** Results of matched multivariable Cox regression on 1–59 month mortality, with shared frailty defined by matched strata

Covariate	Hazard ratio	95% Confidence interval	*P* value
Wealth			
Lowest (ref)	1		
Fourth	1.07	(0.88–1.31)	0.503
Middle	1.10	(0.89–1.35)	0.377
Second	1.12	(0.90–1.39)	0.311
Highest	0.68	(0.51–0.90)	0.007
Child age			
1–5 months (ref)	1		
6–11 months	0.96	(0.79–1.17)	0.664
12–23 months	0.42	(0.34–0.52)	< 0.001
24–35 months	0.29	(0.23–0.37)	< 0.001
36–47 months	0.17	(0.13–0.22)	< 0.001
48–59 months	0.10	(0.07–0.14)	< 0.001
Mother’s age			
15–19 years (ref)	1		
20–24 years	1.01	(0.71–1.44)	0.970
25–29 years	0.85	(0.58–1.24)	0.393
30–34 years	1.18	(0.79–1.78)	0.420
35–39 years	1.16	(0.73–1.85)	0.528
40–44 years	1.12	(0.65–1.95)	0.679
45–49 years	1.03	(0.52–2.03)	0.931
Parity	1.00	(0.95–1.06)	0.894
Diarrhea prevalence	1.65	(0.80–3.38)	0.173
DPT3 coverage (continuous)	0.58	(0.26–1.30)	0.184
*Pf*PR_2–10_ (continuous)	1.00	(1.00–1.00)	0.467
Season (December–May)	0.90	(0.70–1.15)	0.395
Rainfall (lagged 2 months)	1.00	(1.00–1.00)	0.663
Minimum temperature (lagged 2 months)	0.99	(0.97–1.02)	0.502
HH ≥ 1 ITN	0.75	(0.62–0.90)	0.002

ITN = insecticide-treated net; *Pf*PR_2–10_ = *Plasmodium falciparum* prevalence rate in children age 2–10 years.

In analyses stratified by urban/rural residence, there was a larger effect of ITN ownership on mortality in urban areas (HR = 0.32, 95% CI = 0.15–0.66) than in rural areas (HR = 0.80, 95% CI = 0.66–0.97) (data not shown). Additionally, analysis including ITN age categorized as greater than and less than 1.5 years showed that whereas nets reported as younger than 1.5 years were protective (HR = 0.73, 95% CI = 0.60–0.88), older nets were not (HR = 1.12, 95% CI = 0.70–1.97) (data not shown). ITN ownership was associated with lower mortality for younger children (less than or equal to 3 years of age) (HR = 0.78, 95% CI = 0.63–0.97) but not for older children (HR = 1.12, 95% CI = 0.72–1.74). In households with at least one ITN, the number of ITNs was not associated with mortality.

### District-level model.

**Figure 2.**
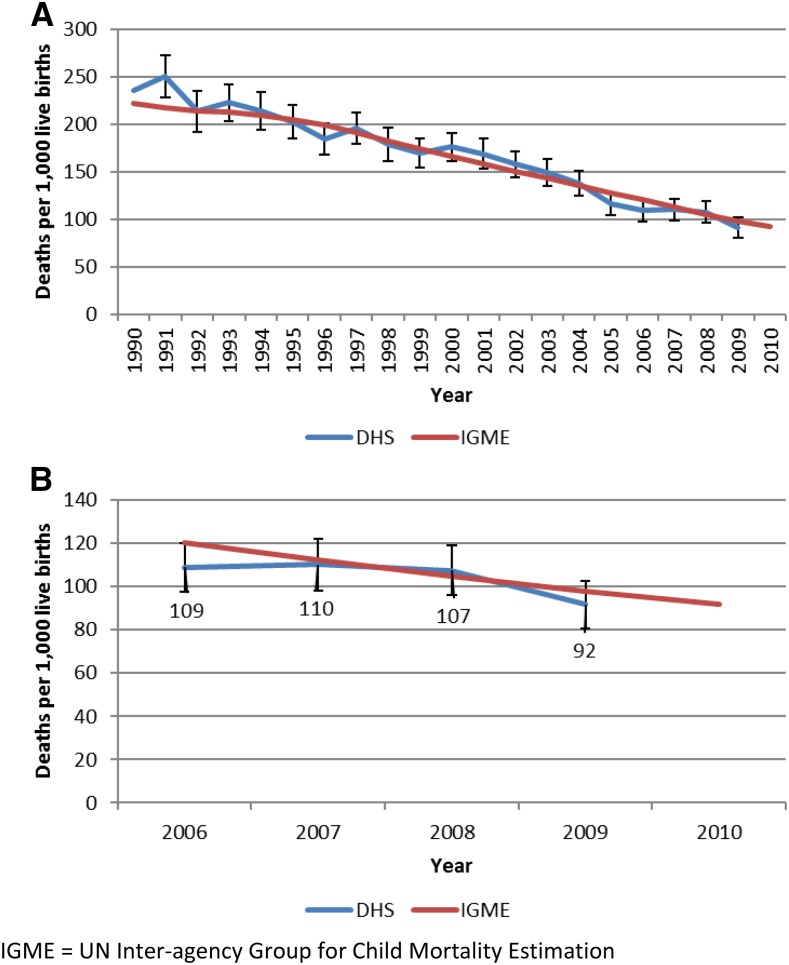
Annual all-cause under-five mortality rate for Malawi. (**A**) From 1990–2010; (**B**) From 2006–2010. IGME = UN Inter-agency Group for Child Mortality Estimation.

Overall, data from 27 districts over a period of 5 years were compiled for use in these analyses for a total of 135 district-years. The 2006 survey reported only aggregated data for two districts, Mwanza and Neno, thus these districts were given the same values for 2006. Likoma and Nkhata Bay were aggregated in both the 2006 and the 2010 surveys due to the small population size of Likoma. District-level under-five annual death estimates ranged from a mean of 17 in 2006 (range: 3–56) and a mean of nine in 2010 (range: 1–31) (Supplemental Table 1). At a national level, under-five mortality declined from 109 to 92 deaths per 1,000 live births between 2006 and 2010 in annual estimates (Figure 2A). The 5-year all-cause under-five mortality rate reported in the 2010 DHS was 112 deaths per 1,000 live births (Figure 2B).

**Figure 3.**
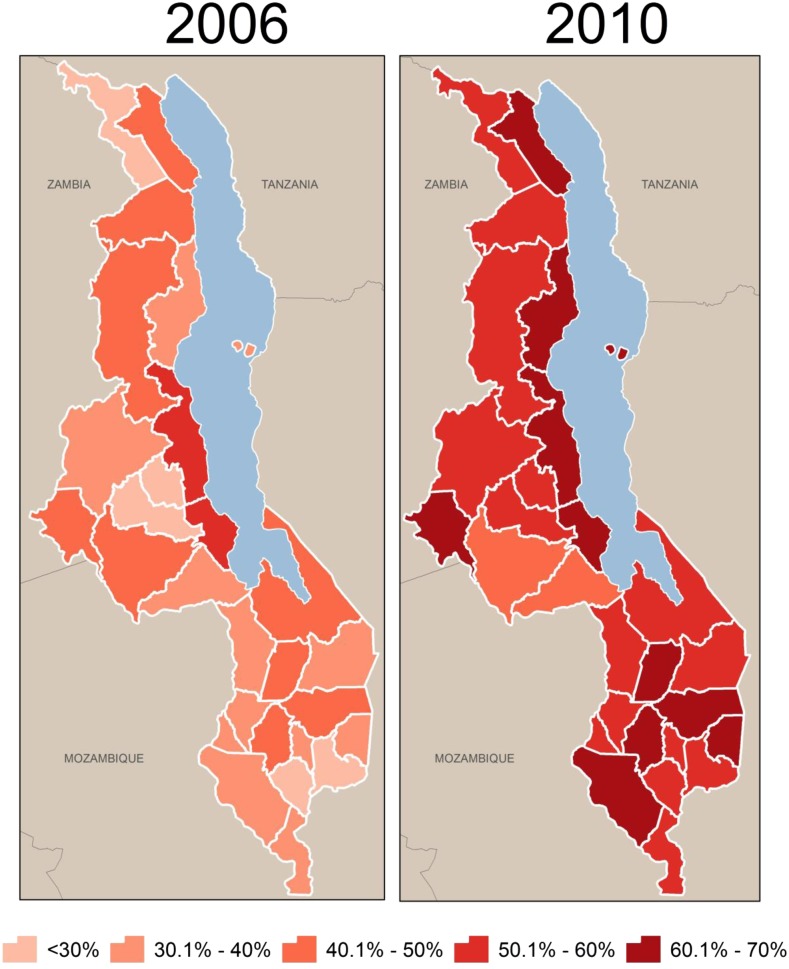
Household ownership of insecticide-treated nets by district, 2006–2010.

**Figure 4.**
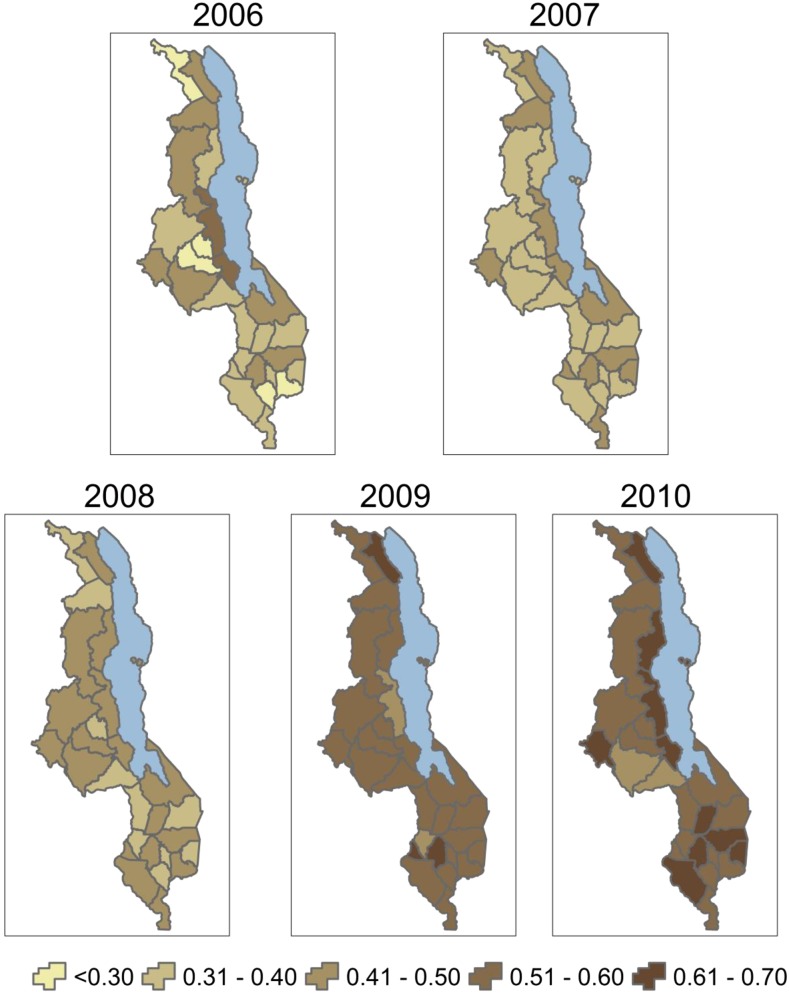
Annual district-level insecticide-treated net (ITN) coverage estimated from ITN ownership data from national surveys and from ITN distribution data adjusted for population and a decay factor, 2006–2010.

In addition to declines in under-five mortality, household ITN ownership has increased significantly over the same period; however, levels of coverage and changes in the coverage over the study period vary by district (Figure 3). Among the study population (children less than five) ITN ownership increased from a district-level average of 36.7% in 2006 (range: 22.3–52.5%) to 69.9% in 2010 (range: 57.9–79.3%) (Table 6). ITN coverage estimated using district-level ITN distribution data were also examined. Modeled ITN coverage increased from 36% (range: 22–53%) in 2006 to 58% (range: 49–67%) in 2010 (Figure 4).

Trends in other sociodemographic variables and in child and maternal health conditions or interventions are summarized in Table 5. Most of the average district-level coverage of demographic and child and maternal health covariates experienced positive change over time (increases for protective interventions and decreases for measures of morbidity). Average, district-level ITN ownership increased the most between 2006 and 2010 from 37% to 70%. Average, district-level percent of women with assisted deliveries increased from 55% to 78% and average, district-level percent of children with vitamin A supplementation increased from 66% to 76% from 2006 to 2010. The average district-level percent of children 12–23 months of age with full immunization increased from 60% to 66% and the average district-level percent of children less than 6 months of age exclusively breastfed increased from 56% to 71% over this period. Smaller increases in district-level averages were seen in mothers with at least a primary school education, access to improved water and sanitation, household wealth and urban residence. Average, district-level prevalence of diarrhea and fever decreased between 2006 and 2010 (23–15% and 35–31%, respectively), as did the proportion of underweight children (19–13%). In contrast, the average district-level proportion of children stunted rose slightly over this time (45–47%) as did the proportion reported to be small at birth (14–15%). The district-level average proportion of women receiving at least two doses of tetanus toxoid during pregnancy decreased from 68% to 61% and the proportion of women attending at least four antenatal care (ANC) visits decreased from 50% to 40%.

**Table 5:** Distribution of variables: mean, minimum and maximum district-level values by year

	2006		2007		2008		2009		2010		Trend
	Mean	(Minimum, Max)	Mean	(Minimum, Maximum)	Mean	(Minimum, Maximum)	Mean	(Minimum, Maximum)	Mean	(Minimum, Maximum)	
Sociodemographic variables											
Urban residence (%)	9.5	(0, 58.6)	9.5	(0, 58.6)	9.8	(0.3, 63.5)	9.8	(0.3, 63.5)	10.2	(0.8, 70.0)	↑
Household wealth (1–5)	2.8	(1.8, 4.2)	2.8	(1.8, 4.2)	2.8	(2.1, 4.1)	2.8	(2.1, 4.1)	2.9	(2.2, 4.1)	↑
Mother primary education (%)	77.1	(49.4, 97.7)	77.1	(49.4, 97.7)	80.5	(58.1, 98.3)	80.5	(58.1, 98.3)	83.9	(61.4, 98.9)	↑
Improved water source (%)	75.6	(57.5, 88.7)	75.6	(57.5, 88.7)	77.9	(63.0, 87.8)	77.9	(63.0, 87.8)	81.1	(63.9, 92.4)	↑
Improved sanitation facilities (%)	1.3	(0, 10.1)	1.3	(0, 10.1)	2.2	(0.2, 7.2)	2.2	(0.2,7.2)	3.1	(0.3, 10.9)	↑
Malaria transmission risk (%)	41.1	(26.9, 55.9)	41.1	(26.9, 55.9)	41.1	(26.9, 55.9)	41.1	(26.9, 55.9)	41.1	(26.9, 55.9)	—
Rainfall anomaly	72.2	(−7.7, 249.5)	115.0	(−57.4, 275.3)	−86.0	(−149.8, 16.3)	−10.7	(−105.4, 142.3)	−3.6	(−160.8, 151.7)	—
Maternal and child health intervertion variables											
Tetanus immunization 2+ (%)	67.9	(50.8, 85.3)	67.9	(50.8, 85.3)	65.0	(49.3, 76.8)	65.0	(49.3, 76.8)	61.2	(38.1, 77.3)	↓
Assisted delivery (%)	54.8	(41.4, 78.5)	54.8	(41.4, 78.5)	66.3	(55.2, 85.9)	66.3	(55.2, 85.9)	77.8	(63.3, 92.7)	↑
Antenatal care 4+ (%)	50.3	(26.0, 67.1)	50.3	(26.0, 67.1)	45.3	(30.7, 55.4)	45.3	(30.7, 55.4)	39.7	(24.0, 58.3)	↓
Immunization status (complete) (%)	59.8	(45.2, 70.1)	59.8	(45.2, 70.1)	62.9	(50.9, 74.9)	62.9	(50.9, 74.9)	66.1	(53.2, 80.1)	↑
Vitamin A supplementation (%)	65.9	(54.8, 80.1)	65.9	(54.8, 80.1)	74.0	(64.4, 81.9)	74.0	0 (64.4, 81.9)	75.5	(65.5, 83.1)	↑
Exclusive breastfeeding < 6 months (%)	56.2	(36.2, 72.1)	56.2	(36.2, 72.1)	64.7	(52.2, 77.0)	64.7	(52.2, 77.0)	70.5	(54.2, 87.8)	↑
Childhood illness variables											
Diarrhea (%)	23.2	(9.64, 32.2)	23.2	(9.64, 32.2)	20.0	(10.5, 28.8)	20.0	(10.5, 28.8)	15.4	(5.76, 25.0)	↓
ARI (%)	7.4	(2.0, 13.7)	7.4	(2.0, 13.7)	7.0	(4.1, 11.9)	7.0	(4.1, 11.9)	6.1	(2.0, 12.1)	↓
Fever (%)	34.8	(18.3, 53.4)	34.8	(18.3, 53.4)	34.2	(19.9, 49.3)	34.2	(19.9, 49.3)	30.7	(16.6, 50.5)	↓
Stunting (%)	45.2	(29.7, 57.0)	45.2	(29.7, 57.0)	46.3	(33.7, 55.2)	46.3	(33.7, 55.2)	47.3	(37.7, 58.1)	↑
Underweight (%)	19.4	(13.0, 29.2)	19.4	(13.0, 29.2)	16.2	(10.0, 22.3)	16.2	(10.0, 22.3)	13.0	(5.97, 23.5)	↓
Small size at birth (%)	14.4	(7.9, 21.1)	14.4	(7.9, 21.1)	14.7	(10.1, 26.0)	14.7	(10.1, 26.0)	15.5	(7.15, 37.0)	↑
Malaria control intervention variables											
ITN coverage high (%)	18.5	(0, 100)	18.5	(0, 100)	63.0	(0, 100)	51.9	(0, 100)	14.8	(0, 100)	—
ITN ownership (household) (%)	36.7	(22.3, 52.5)	36.7	(22.3, 52.5)	47.2	(37.6, 58.7)	47.2	(37.6, 58.7)	69.9	(57.9, 79.2)	↑

ARI = acute respiratory infection; ITN = insecticide-treated net.

In negative binomial models higher ITN ownership was marginally significantly associated with lower all-cause under-five mortality (incidence rate ratio = 0.50; 95% CI = 0.25–1.02) controlling for year, and measures of wealth, urban residence, access to improved water and sanitation, mothers’ education, mothers’ tetanus immunization coverage, children’s immunization coverage, vitamin A supplementation coverage, stunting in children, diarrhea prevalence, malaria transmission risk, and rainfall anomalies (Table 6). Mortality in children under five decreased over time in these models between 2006 and 2008 but was not significantly lower in 2009 or 2010 compared with 2006. Under-five deaths were significantly less likely in districts in which a greater percent of children received vitamin A supplementation than in districts with lower vitamin A coverage (incidence rate ratio [IRR] = 0.85; 95% CI = 0.74–0.97). Against expectations, under-five deaths were higher in districts with higher proportion of households having access to improved water sources (IRR = 1.16; 95% CI = 0.98–1.37) and in districts with higher coverage of tetanus immunization in mothers (IRR = 1.18; 95% CI = 1.05–1.32).

**Table 6:** Results of negative binomial regression model of deaths in children less than 5 years old in Malawi 2006–2010: ITN ownership as exposure

	IRR	LCI	UCI	*P* value
Year				
2006	1			
2007	0.83	0.71	0.96	0.015
2008	0.78	0.64	0.95	0.014
2009	0.99	0.81	1.22	0.946
2010	0.80	0.59	1.08	0.14
Percent of children in households owning at least one ITN				
Low (ref)	1			
High	0.88	0.76	1.02	0.085
Percent of children living in wealthier households				
Low (ref)	1			
High	0.95	0.81	1.10	0.467
Percent of children with access to improved water sources				
Low (ref)	1			
High	1.16	0.98	1.37	0.08
Percent of children whose mothers have at least a primary education				
Low (ref)	1			
High	0.94	0.75	1.17	0.571
Percent of children 6−59 months stunted				
Low (ref)	1			
High	1.05	0.94	1.16	0.395
Percent of children whose mothers had two doses of tetanus immunization				
Low (ref)	1			
High	1.18	1.05	1.32	0.004
Percent of children living in urban residences				
Low (ref)	1			
High	0.96	0.78	1.17	0.681
Percent of children with improved sanitation facilities				
Low (ref)	1			
High	1.09	0.91	1.31	0.331
Percent of children 12–23 months fully immunized				
Low (ref)	1			
High	1.13	0.87	1.46	0.366
Percent of children less than 5 with diarrhea in the past 2 weeks				
Low (ref)	1			
High	1.10	0.97	1.26	0.15
Percent of children with vitamin A supplementation				
Low (ref)	1			
High	0.85	0.74	0.97	0.018
*Pf*PR_2–10_				
Low (ref)	1			
High	0.99	0.87	1.13	0.881
Rain anomalies				
Low (ref)	1			
High	0.93	0.80	1.08	0.316

IRR = incidence rate ratio; LCI = lower confidence interval; UCI = upper confidence interval; BIC = −452.7889; Pearson deviance = 1.02809

Models run with district-level modeled household ITN ownership showed similar significant results (modeled ITN ownership was associated with declines in mortality, IRR = 0.77; 95% CI = 0.60–0.98) indicating that findings were robust across several different measures of ITN ownership (Table 7).

**Table 7:** Results of negative binomial regression model of deaths in children less than 5 years old in Malawi 2006–2010: modeled ITN ownership as exposure

	IRR	LCI	UCI	*P* value
Year				
2006	1			
2007	0.82	0.70	0.96	0.011
2008	0.78	0.63	0.96	0.022
2009	1.24	0.87	1.75	0.231
2010	0.88	0.62	1.25	0.476
Percent of children in households owning at least one ITN				
Low (ref)	1			
High	0.77	0.60	0.98	0.035
Percent of children living in wealthier households				
Low (ref)	1.00			
High	0.94	0.81	1.09	0.403
Percent of children with access to improved water sources				
Low (ref)	1			
High	1.13	0.95	1.36	0.168
Percent of children whose mothers have at least a primary education				
Low (ref)	1			
High	0.94	0.75	1.19	0.619
Percent of children 6–59 months stunted				
Low (ref)	1			
High	1.07	0.97	1.19	0.184
Percent of children whose mothers had two doses of tetanus immunization				
Low (ref)	1			
High	1.17	1.04	1.32	0.01
Percent of children living in urban residences				
Low (ref)	1			
High	1.01	0.82	1.23	0.956
Percent of children with improved sanitation facilities				
Low (ref)	1			
High	1.02	0.87	1.21	0.785
Percent of children 12–23 months fully immunized				
Low (ref)	1			
High	1.18	0.92	1.53	0.198
Percent of children less than 5 with diarrhea in the past 2 weeks				
Low (ref)	1			
High	1.10	0.96	1.27	0.173
Percent of children with vitamin A supplementation				
Low (ref)	1			
High	0.81	0.72	0.91	0.001
*Pf*PR_2–10_				
Low (ref)	1			
High	0.99	0.85	1.14	0.854
Rain anomalies				
Low (ref)	1			
High	0.93	0.79	1.09	0.372

IRR = incidence rate ratio; LCI = lower confidence interval; UCI = upper confidence interval. BIC = −458.1381; Pearson deviance = 1.020938.

## DISCUSSION

All-cause under-five mortality has rapidly declined over the past several decades in Malawi. The 1992 Malawi DHS estimated 234 under-five deaths for every 1,000 live births for the 5-year period preceding the survey. By 2010, this number was halved, to 112 under-five deaths. In our study, results from both individual-level and district-level regression models suggest that ownership of ITNs in the period preceding the 2010 DHS was associated with declines in all-cause under-five mortality in Malawi from 2006 to 2010. The estimated relative reduction in child mortality ranged from 23% to 50%, depending on the model, which is consistent with the magnitude of protective effects shown in randomized control trials (17% comparing ITNs to no nets).^4^ Results are also consistent with those of other country-specific observational studies including a cohort study from Kenya in which ITN use was associated with a 44% relative reduction in mortality among children 1–59 months of age (95% CI = 4–67%),^5^ a case-control study from Tanzania in which the estimated protective efficacy of ITN use was 27% (95% CI = 3–45%) among children 1 month to 4 years of age,^6^ and a postdistribution evaluation in the Gambia in which the protective efficacy (PE) of ITN use among children 1–9 years of age was 25% (95% CI = 2–43%).^7^ All of these analyses have been conducted in settings of high malaria endemicity and across a range in levels of ITN use.

Demonstrating the benefits of ITN use on malaria-specific outcomes would be preferable to studies of all-cause mortality. Eisele and others estimated the protective efficacy of ITNs on reducing malaria-attributable mortality in children 1–59 months to be as much as 55% (49–61%) in a meta-analysis of RCTs.^26^ However, reliable cause-specific mortality data are often not available or not reliable. Many deaths occur outside of the formal health system and the available tools for capturing cause of death from communities, such as verbal autopsy, have been shown to have low sensitivity and specificity for identification of malaria deaths.^27,28^ Given these limitations, and the research showing that the indirect effects of malaria may in fact double the malaria-attributable mortality estimates,^29^ all-cause childhood mortality has been found to be a valid indicator of malaria-specific mortality in countries with high, stable malaria transmission.^30^

Results of this study contribute to a small body of evidence showing protective effects of ITNs on all-cause childhood mortality under routine conditions at a national level. Lim and others conducted an observational study pooling data from 29 nationally representative household surveys and found an overall protective effect of 23% (13–31%).^8^ Data from the Malawi 2004 DHS were included by Lim and others but a significant protective effect was not found. It is possible that the large sample size in the Malawi 2010 DHS lent the analysis enough power to observe the protective effect of ITNs that is otherwise often elusive in analyses of survey data. In fact, protective effects were seen in only five of the 29 surveys when analyzed independently. The lack of effect may be largely due to limitations of survey data, mainly that the cross-sectional data limit the ability to establish causal associations between ITN ownership and health outcomes.

In this study, several approaches were used to account for the limitations associated with cross-sectional data. Individual-level analyses used the cross-sectional data on the length of time a household has owned each bednet from the net roster, included in household questionnaires, to create a retrospective cohort. This longitudinal dataset included information on child births and deaths as well as ITN ownership over the 36 months preceding the survey thus permitting longitudinal survival analysis to be done. Additionally, the use of exact matching limited confounding by comparing the survival outcomes of ITN owners and nonowners in the same matched strata. This analytic technique improved our ability to attribute observed associations to causal relationships. Also, considerable progress in the scale-up of ITNs had occurred between 2006 and 2010, permitting a trend analysis of ITN impact on child mortality.

Although a creative approach to the shortfalls of survey data, these longitudinal models have some limitations of their own. The data collected on duration of ITN ownership depends entirely on recall from the interviewed head of household about when nets were obtained for a period of up to 3 years. Even if accurate recall is possible over this period, data are collected only for existing nets at the time of the survey, and not about any nets that were owned during the 3 year period but discarded at some point preceding the survey. Assuming accurate recall, ITN ownership is likely to be underestimated using this technique. This measure of ITN ownership is also not specific to the child whose mortality risk is being estimated in the models as the metric does not measure retrospective use of ITNs by individual children. In fact, ITN use was reported by only 57% of children under-five in households owning at least one ITN in the Malawi 2010 DHS, which suggests that misclassification bias of ITN exposure may influence results. However, this misclassification may be somewhat mitigated by the insecticidal properties of ITNs which confer a community-level protective effect even on those who do not sleep under the net. In addition, the covariates available for inclusion in models are limited to cross-sectional variables from the survey, which may not be accurate estimates for a point in time 3 years before. Many of these variables, such as immunization coverage, were not available for the children who had died necessitating aggregation at the cluster-level. Finally, while exact matching and the inclusion of household wealth as covariate reduces the potential for confounding due to factors such as socioeconomic status, it does not remove it entirely, and in a subanalysis of households with at least one ITN, we were unable to detect an association between the number of ITNs owned and child mortality.

District-level regression models were used in this study as a second approach to overcoming the challenges of using cross-sectional survey data for determination of causal relationships. Despite the call for district-level platform analyses for impact evaluations by Victora and others,^9^ surveys sampled at the district level are not common. The sample size requirements for district-level representation of health indicators require substantially more funds than surveys sampled at lower spatial levels. The benefits of having access to district-level data are not often found to be cost-effective. Due to the paucity of nationally representative household surveys sampled at the district-level, examples of similar district-level models are few. Several studies from Eritrea^10^ and Zambia^11^ used district-level, facility-based, routine data from Health Management Information Systems (HMIS) to look at the effects of malaria interventions on malaria cases. Using longitudinally collected HMIS data permits use of analytic tools that allow more robust assessments of causal relationships between interventions and malaria-specific outcomes. Strengthening the systems to provide these multiple data sources is an approach the PMI now advocates for to facilitate future impact evaluations.

One advantage of the district-platform analytic approach over the individual-level model is the ability to use population-level retrospective exposure data (ITN ownership) from surveys and/or from program ITN distribution data without having to make assumptions based on current net ownership. Another advantage is that data on other important covariates can be estimated for every district for every year. Malawi is unusual in that more than one nationally representative survey was completed with district-level sampling. This permitted a greater number of parameters to be entered into the model. Although this process requires extrapolation of estimates from survey data and assumptions of linear changes over time, it permits the control of important predictors of child mortality in the analyses. In this case, in addition to ITN ownership, district-year measures of tetanus immunization coverage in mothers, and vitamin A supplementation in children were also found to be important predictors of child mortality over the study period.

Despite the promising possibilities afforded by the district-level models used in this study, there are several limitations. First, temporal ambiguity affects interpretation of results; exposure to ITNs or other interventions are estimated for an annual period and may not have preceded the deaths that are also aggregated annually. Second, these are ecologic models that could be affected by the ecologic fallacy, specifically that relationships observed at the aggregate level may not hold at the individual level. Finally, despite the very large sample size of the 2010 DHS, the data are condensed into district-years for these analyses, leaving a sample size of only 135. This small sample size affects the stability of the estimates. Perhaps most significantly, the models did not account for spatial correlation that may be occurring at the district level. Temporal correlation was accounted for using robust clustering commands in Stata. Future efforts could be made to test and adjust for spatial correlation using Bayesian modeling techniques.

This study demonstrates that publically available nationally representative data can be used to examine impact of malaria control interventions under routine conditions. The analytic approaches used in this study could be applied to estimate ITN impact disaggregated by age or by exposure history. Given that both of the approaches used in this study to estimate the effects of ITN ownership on all-cause child mortality (ACCM) are limited by the constraints of cross-sectional survey data, identification of alternative, nationally representative data, or other creative analytic approaches must be a priority for the future. Even with few surveys being sampled at district-level spatial scales, it is becoming increasingly possible to use high resolution surfaces of indicators such as ITN coverage, malaria parasitemia prevalence and other demographic and health indicators created from survey data to construct datasets for analysis. However, operationalization of the data into analyses needs further exploration and uncertainty around estimates should be carefully considered in this type of analysis. Longitudinal morbidity and mortality data stemming from health facilities and vital registration systems may become more useful as reporting systems are strengthened.

## CONCLUSION

ITN ownership was found to be significantly associated with decreased risk of dying from all-causes in children less than 5 years of age, both at the individual- and at the district-level, adjusting for other available predictors of child survival. The mortality risk decreased as children progressed toward 5 years of age. The protective effect of ITN ownership on child survival was robust across several different approaches for modeling ITN ownership and the magnitude of effect was in line with estimates from other published studies. The results of this study provide evidence to support the impact of malaria control intervention expansion on reductions in ACCM in Malawi.

## Supplementary Material

Supplemental Table.
