# Supplementary material for: Impact of Insecticide-Treated Net Ownership on All-Cause Child Mortality in Malawi, 2006–2010
Source: Am J Trop Med Hyg. 2017 Sep 27;97(3 Suppl):65–75. doi: 10.4269/ajtmh.15-0929 (PMC5619930; doi:10.4269/ajtmh.15-0929)
Supplement: Supplementary file 1 [file tpmd150929.SD1.pdf]

The following are supplemental materials and will be published online only

doi:10.4269/ajtmh.15-0929.s001

SUPPLEMENTAL APPENDIX

The rapid expansion of malaria control interventions in Malawi did not occur in a vacuum. Many other concomitant changes in economic, social, and health factors were also underway. Results from this analysis found that risk of under-five death was lower in districts with greater coverage of vitamin A supplementation in children less than 5 years of age. Vitamin A coverage was measured for the 6 months preceding each survey. The direction of effect was counterintuitive for coverage of tetanus immunization in women. Children living in districts in which a large proportion of the women with births in the past 2 years received at least two tetanus immunizations had higher mortality risk than those from districts with low tetanus immunization coverage in women. As tetanus immunizations are administered during ANC visits, it could be that women with higher risk pregnancies are more likely to interface with formal health-care providers during pregnancy and therefore are more likely to receive two doses of tetanus immunization.

Interestingly, rainfall and $Pf\mathrm{PR}_{2-10}$ were not significant predictors of child mortality in either the individual-level or the district-level multivariable model. It is possible that the climate of Malawi over the periods investigated is not sufficiently heterogeneous for these factors to affect the association between ITNs and child mortality. Similarly, $Pf\mathrm{PR}_{2-10}$ is a temporally static value representative of the baseline malaria transmission. There is also little spatial variation in $Pf\mathrm{PR}_{2-10}$ in Malawi when aggregated to the district level. It is therefore not surprising that no association is seen with child mortality given the lack of heterogeneity in the measure and the many other causes of child mortality. Bennett and others used model-based geostatistical methods to look at change in $Pf\mathrm{PR}_{2-10}$ in Malawi between 2000 and 2010 and found no evidence of a reduction.[31] This could be due in part to the role of climatic factors that led 2010 to have higher prevalence than expected. It could also be due to the lack of sensitivity of $Pf\mathrm{PR}_{2-10}$ to detect meaningful changes in force of infection and/or transmission in settings of medium to high transmission. For example, Giardina and others used spatial and temporal analysis to look at the effects of vector-control interventions on malaria parasitemia in sub-Saharan Africa (SSA).[32] They found variation in the association between intervention coverage and parasitemia risk between and within countries. In some regions no decline in parasitemia prevalence was detected despite significant intervention effects.

Supplemental Table 1

Survey weighted counts of deaths of children less than 5 years of age and sample population at the district level using data from birth histories from the 2010 DHS*

	2006		2007		2008		2009		2010	
District	Deaths	Population	Deaths	Population	Deaths	Population	Deaths	Population	Deaths	Population
Chitipa	3	1,304	4	1,363	2	1,389	1	1,413	2	1,005
Karonga	3	1,289	6	1,386	2	1,440	6	1,470	3	1,110
Nkhatabay	5	1,246	7	1,275	3	1,291	3	1,279	2	947
Rumphi	3	1,297	4	1,365	3	1,404	3	1,391	2	956
Mzimba	52	1,487	22	1,552	32	1,593	23	1,568	14	1,114
Kasungu	27	1,546	25	1,661	21	1,681	39	1,731	10	1,225
Nkhota kota	6	1,511	10	1,574	11	1,587	9	1,563	7	1,209
Ntchisi	7	1,317	6	1,393	3	1,458	6	1,436	1	1,030
Dowa	7	1,215	9	1,331	18	1,385	18	1,386	6	1,021
Salima	16	1,314	15	1,390	17	1,396	15	1,459	3	990
Lilongwe	56	1,622	57	1,677	51	1,705	56	1,719	31	1,205
Mchinji	14	1,474	16	1,546	10	1,579	22	1,600	11	1,153
Dedza	21	1,393	23	1,437	22	1,476	44	1,453	18	1,005
Ntcheu	18	1,438	26	1,506	17	1,468	19	1,405	7	1,042
Mangochi	30	1,325	29	1,426	18	1,508	51	1,539	17	1,110
Machinga	15	1,443	10	1,488	15	1,560	17	1,546	8	1,158
Zomba	19	1,334	21	1,440	25	1,505	26	1,484	11	1,034
Chiradzulu	10	1,198	9	1,228	7	1,247	6	1,182	7	920
Blantyre	39	1,554	27	1,579	30	1,590	23	1,566	21	1,197
Mwanza	3	1,274	2	1,309	2	1,323	2	1,266	1	919
Thyolo	16	1,403	11	1,473	10	1,528	19	1,417	5	976
Mulanje	17	1,395	25	1,443	21	1,440	16	1,394	14	985
Phalombe	13	1,521	8	1,656	14	1,693	11	1,704	6	1,151
Chikwawa	29	1,417	18	1,446	19	1,545	21	1,515	20	1,187
Nsanje	11	1,382	7	1,458	9	1,497	10	1,512	5	1,126
Balaka	10	1,378	8	1,395	8	1,448	16	1,465	4	1,035
Neno	3	1,228	2	1,290	2	1,311	2	1,290	1	921
Total	453	37,305	407	39,087	392	40,047	484	39,753	237	28,731

*These are weighted estimates of under-five deaths.
